# Correction: REM-related obstructive sleep apnea and vertigo: A retrospective case-control study

**DOI:** 10.1371/journal.pone.0303980

**Published:** 2024-05-16

**Authors:** Po-Yueh Chen, Tzu-Ying Chen, Pin-Zhir Chao, Wen-Te Liu, Chyi-Huey Bai, Sheng-Teng Tsao, Yi-Chih Lin

There are errors in the author affiliations. The correct affiliations are as follows:

Po-Yueh Chen^1,2,3^, Tzu-Ying Chen^4^, Pin-Zhir Chao^3,4^, Wen-Te Liu^3,5^, Chyi-Huey Bai^6^, Sheng-Teng Tsao^6^, Yi-Chih Lin^2,3,4,6^

**1** Department of Otolaryngology, Wan Fang Hospital, Taipei Medical University, Taipei, Taiwan, **2** Department of Otolaryngology, School of Medicine, College of Medicine, Taipei Medical University, Taipei, Taiwan, **3** Sleep Centre, Shuang Ho Hospital, Taipei Medical University, New Taipei City, Taiwan, **4** Department of Otolaryngology, Shuang Ho Hospital, Taipei Medical University, New Taipei City, Taiwan, **5** Department of Chest, Shuang Ho Hospital, Taipei Medical University, New Taipei City, Taiwan, **6** Department of Public Health, College of Medicine, Taipei Medical University, Taiwan
