# Supplementary material for: REM-related obstructive sleep apnea and vertigo: A retrospective case-control study
Source: PLoS One. 2021 Jun 11;16(6):e0252844. doi: 10.1371/journal.pone.0252844 (PMC8195434; doi:10.1371/journal.pone.0252844)
Supplement: Attachment — Submitted filename: Revisor response.docx [file pone.0252844.s002.docx]

Dear Editor,

It was a pleasure to collaborate in this revision. The manuscript appears well written and structured. However, some minor corrections could improve the overall quality.

Maybe more fascinating title would be: REM-Related Obstructive Sleep Apnea and Vertigo: a Retrospective case-control study.

**Introduction**

- Add reference line 1 of vertigo prevalence.
- ‘’A national Taiwan study’’ insert p value data.
- ‘’ In recent population-based case–control studies’’Describe data of the population analyzed in the study to highilight the connection and add this reference: doi: 10.5664/jcsm.5080.
- ‘’ OSA is a frequent disorder characterized by’’ also cite other classical sleep stage in children and elderly patients using these references: doi:10.1164/ajrccm.162.2.9908058; DOI: [10.20452/pamw.15283](https://doi.org/10.20452/pamw.15283) ; DOI: [10.1093/sleep/30.7.837](https://doi.org/10.1093/sleep/30.7.837)

**Study design**

- Cite AASM guidelines for PSG describing better the procedures

**Ethical approval** is written in red: modify

**Tables** describe in wich group was performed the analysis: total vs control, total vs vertigo or what?

In the captations specify: Abbreviations: st90, sleep time…

**Discussion** PSG index is not anymore considered the only effective tool to evaluate sleep breathing disorder especially in children. Describe a different procedure and the data reported in this study doi: 10.1016/j.ijporl.2017.04.021.

**Minor corrections:**

**Introduction**

- correct ‘’were experiencing’’;
- modify center in centre;
- correct tumor in tumour;
- who were blindend in who was blinded;
- t test in t-test;
- or exinergic activity in orexinergic activity;

**Discussion**

- correct ‘’tends’’ in tended
